# Reliability of Endolymphatic Hydrops Qualitative Assessment in Magnetic Resonance Imaging

**DOI:** 10.3390/jcm12010202

**Published:** 2022-12-27

**Authors:** Emilia Wnuk, Magdalena Lachowska, Agnieszka Jasińska-Nowacka, Edyta Maj, Kazimierz Niemczyk

**Affiliations:** 1 2nd Department of Clinical Radiology, Medical University of Warsaw, 02-097 Warsaw, Poland; 2 Department of Otorhinolaryngology Head and Neck Surgery, Medical University of Warsaw, 02-097 Warsaw, Poland

**Keywords:** endolymphatic hydrops, inner ear, magnetic resonance imaging, Ménière’s disease, vertigo, 3D-FLAIR

## Abstract

The study aimed to compare the consistency of MRI interpretation of endolymphatic hydrops qualitative assessment of inner ear structures performed by independent observers. MRI with a delayed post-contrast 3D-FLAIR sequence was performed to visualize EH in patients suspected of having or diagnosed with MD. The scans were analyzed independently by three observers. In total, 220 ears were evaluated and, of these, 75 had definite MD, five probable MD, 67 with other Menieriform symptoms, and 73 were asymptomatic. Significant differences in cochlear endolymphatic hydrops (CoEH) grading between all observers were observed. On the Barath scale of vestibular endolymphatic hydrops (VEH), differences were found between the radiologists and otorhinolaryngologist in grading. No differences were noted in VEH on the Bernaerts scale and increased perilymphatic enhancement. Our study showed that evaluation of vestibular endolymphatic hydrops is repeatable between observers and easy to learn. It proved that Bernaerts’ modification increased the sensitivity of EH diagnosis. Both parameters, CoEH and VEH, may serve as a differentiation method of EH from normal ears. The distinction between normal and hydropic ears is much easier to perform than EH grading. Therefore, it may be used to diagnose MD rather than EH staging.

## 1. Introduction

Ménière’s disease (MD) is a chronic disorder of the inner ear characterized by spontaneous attacks of vertigo, fluctuating sensorineural hearing loss, tinnitus, and aural fullness [1,2].

The MD diagnosis depends on the patient’s symptoms, medical history, and functional inner ear test results. Since the symptoms of MD are heterogenous and it takes time to develop from monosymptomatic to fully symptomatic disease, diagnosing early stages of MD or atypical forms of the disease is often complicated.

Therefore, scientists have been searching for a distinct characteristic of this disease. Approximately 80 years ago, a hydropic enlargement of the endolymphatic structures (cochlear duct within cochlea, utricle, and saccule of the vestibule) in temporal bone specimens of patients with MD was observed [3]. Even though it was an important discovery, its usefulness was limited, as it was impossible to visualize inner ear structures in vivo. Consequently, the diagnosis of MD has been based on the patient’s symptoms, clinical findings, and functional inner ear test results. The only role of radiology was to exclude other pathologies with symptoms similar to MD [1,2]. With the breakthrough discovery of Nakshima and Naganawa that, in magnetic resonance imaging (MRI) using the delayed post-gadolinium contrast sequences, perilymphatic structures enhance and endolymphatic structures do not, visualizing endolymphatic hydrops (EH) became possible [4]. Since then, many studies have been performed to evaluate if an endolymphatic hydrops is a biomarker of MD or if there are any other imaging features characteristic of this disease. A few imaging methods for the assessment of EH have been described in the literature, as follows: semiquantitative scale [5], qualitative [6], comparison of the size of saccule and utricle [7], and a modification of the qualitative scale by adding an extra-low vestibular hydrops grade [8].

Furthermore, a more robust enhancement of the affected inner ear structures has been reported in MD ears as a sign of blood–labyrinth barrier breakdown [8]. Many studies have described the presence of EH and increased perilymphatic enhancement in MD patients. However, a discrepancy in the reported frequency of EH exists, potentially due to the radiological criteria used.

One of the critical features of imaging in medicine is its reliability concerning image reading. It should be reliable regardless of the observers involved. In clinical settings, usually, it is one expert observer who studies a large number of images (cases), rather than multiple observers studying each case and comparing their results to determine the final image result. In the outpatient department setting, an otolaryngologist takes patients’ history and examines and reads the MRI scans bought to the visit by the patient (sometimes without a result written by a radiologist).

The described frequency of endolymphatic hydrops differs among studies, as mentioned above. Some authors described the presence of endolymphatic hydrops also in healthy patients. It is not clear if endolymphatic hydrops is present only in Ménière’s disease ears or might be present also in other inner ear diseases. This is similar to the asymmetry of perilymphatic enhancement. The debate is still going on, as to whether endolymphatic hydrops and increased perilymphatic enhancement can be biomarkers of Ménière’s disease. From a clinical point of view, it is interesting and important to investigate if the differences between studies presented in the literature might result from MRI scan interpretation by different observers.

This study aimed to evaluate the qualitative assessment of inner ear hydrops to verify if it is consistent between observers and easy to learn from a practical clinical point of view for the needs of the daily work of a radiologist and otolaryngologist in clinical and outpatient department settings. In addition, all the MRI-assessed endolymphatic hydrops features were analyzed to calculate the sensitivity and specificity of the method.

In this study, we performed an MRI interpretation of endolymphatic hydrops qualitative assessment of inner ear structures by independent observers and compared the consistency of their MRI interpretation. We wanted to perform this investigation mimicking real clinical and outpatient department settings; that is, each of the observers interpreted the MRI scan once, and their results were compared. For that reason, three observers were engaged in this study, two radiologists and one otorhinolaryngologist. In addition, engaging an otorhinolaryngologist aimed to check if the evaluated method of endolymphatic hydrops assessment was easy to learn after reasonably short training, so that an ENT specialist (otorhinolaryngologist) in the outpatient department could read inner ear MRI scans by themselves during a patient’s visit.

## 2. Materials and Methods

### 2.1. Ethical Consideration

This prospective study protocol was reviewed and approved by the Institutional Ethics Committee, where the study was conducted (KB/110/2019). All patients gave their written informed consent for participation in the study. The project conforms to The Code of Ethics of the World Medical Association (Declaration of Helsinki).

### 2.2. Study Group Description

Between June 2018 and December 2020, 120 consecutive patients suspected of having or diagnosed with MD according to AAO–HNS and Barany Society criteria [1,2] underwent MRI with delayed post-gadolinium 3D-FLAIR sequence. Furthermore, out of this group, ten patients were excluded due to insufficient clinical information (*n* = 4), insufficient MRI quality (*n* = 3), or previous inner ear surgery (*n* = 3). Eventually, 110 patients were enrolled in this study and underwent a detailed analysis.

### 2.3. Magnetic Resonance Imaging Procedure and Analysis

The patients’ MRI scans were performed using a 3 Tesla MR scanner (Signa Architect, GE Healthcare, Milwaukee, WI, USA) with a 16-channel phased array flex coil (GEM Flex Large coil, Neocoil, Pewaukee, WI, USA). This coil was used to minimize the distance between the inner ear structures and the coil. The examination was carried out in a supine position. A four hours delayed post-contrast axial 3D-FLAIR sequence was added to the standard MRI protocol of the posterior cranial fossa to visualize the inner ear structures. The following parameters characterized the sequence: 3D-FLAIR with a fat-suppression, acquired on an axial plane, covering the posterior cranial fossa; time of repetition 7602 ms, time of echo 170 ms, time of inversion 1897 ms, NEX 2.0, the field of view 18, slice thickness 0.8 mm, variable flip angle. A double dose (0.2 mL/kg) of intravenously injected gadobutrol (Gadovist; Bayer Schering Pharma AG, Berlin, Germany; 1.0 mmol/mL) was used to achieve optimal perilymphatic enhancement [4,6,9]. The posterior cranial fossa protocol also consisted of a three-dimensional fast inflow steady-state acquisition (3D-FIESTA) sequence to visualize the inner ear fluid space.

The MRI scans were analyzed independently by two (head and neck and neuro-) radiologists—(Rad 1 and Rad 2) and an otorhinolaryngologist in training (Oto), who was taught for a short time how to read the inner ear structures on MRI scans. All of them interpreted MRIs independently from one another, and all were blinded to the patients’ clinical status and other diagnostics test results. The goal was to check if the method is repeatable between the different observers and if it is easy to learn.

The following radiological features were assessed in four steps:

1. Cochlear endolymphatic hydrops (CoEH) in a three-stage grading system by Barath [6]
2. Vestibular endolymphatic hydrops (VEH) in a three-stage grading system by Barath [6]
3. VEH in a modified four-stage scale by Bernaerts [8]
4. Enhancement of the inner ear structures [8].

The assessment of the CoEH was performed at the mid-modiolar level, and the VEH was evaluated at the level of the inferior part of the vestibule as described previously [6]. The signal intensity of perilymphatic structures enhancement (PE) was evaluated visually, and any asymmetry between two inner ear structures was reported. Each of the four radiological features was presented on an ordinal scale.

**Figure 1:**
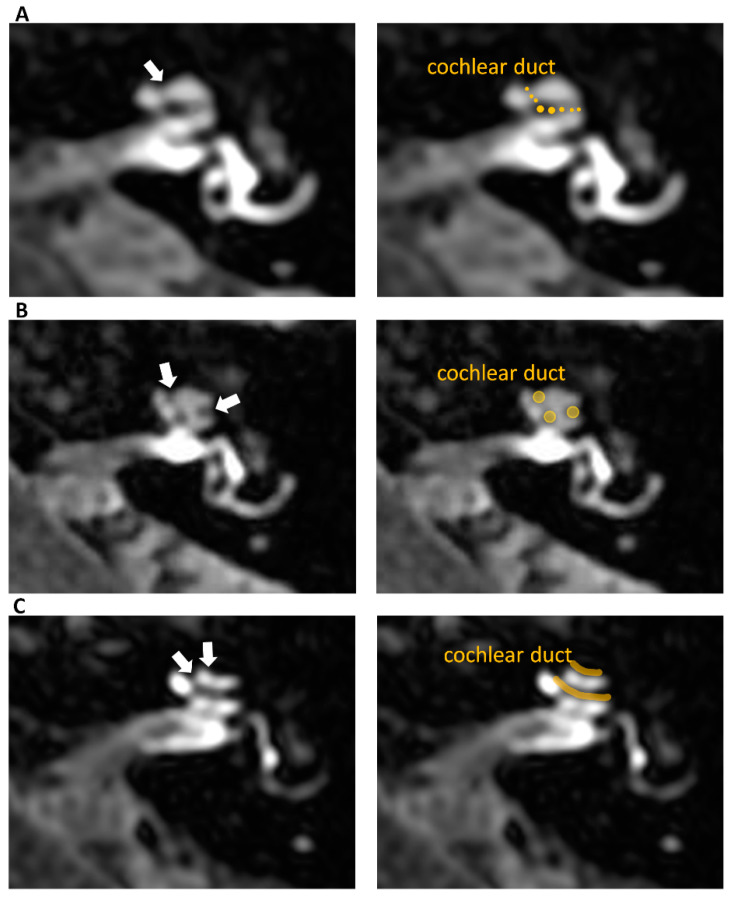
Delayed post-contrast 3D-FLAIR axial images of the left ears at the level of the cochlear modiolus. Right side images on panels (**A**–**C**) show the same images as the left ones in respective panels but in the form of companion scans with some line art applied to define specific anatomical structures for easier identification of observed pathologies. Panel (**A**) presents normal cochlea—grade 0 according to Barath classification, where a non-enhancing cochlear duct is barely visible between the enhancing scala vestibuli and scala tympani (arrow), and no signs of cochlear endolymphatic hydrops are detectable. Panel (**B**) shows grade 1 cochlear endolymphatic hydrops with partial obstruction of the scala vestibuli by a mildly dilated cochlear duct. The scan shows the nodular non-enhancing regions visible at the margins of the cochlea (arrows). Panel (**C**) presents grade 2 cochlear endolymphatic hydrops where the cochlear duct is significantly enlarged and obstructs scala vestibuli. The scan shows the non-enhancing stripes within the enhancing scala tympani (arrows).

*Cochlear endolymphatic hydrops* in the three-stage grading system by Barath [6] was defined as either 0 as normal, 1 or 2 as pathology (Figure 1). Normal indicates a situation where the cochlear duct is faintly visible between the enhancing scala tympani and scala vestibuli. Grade 1 is a moderate cochlear duct enlargement and narrowed scala vestibuli. Grade 2 indicates a significantly enlarged cochlear duct that entirely obliterates scala vestibuli (virtually obstructing scala vestibuli).

**Figure 2:**
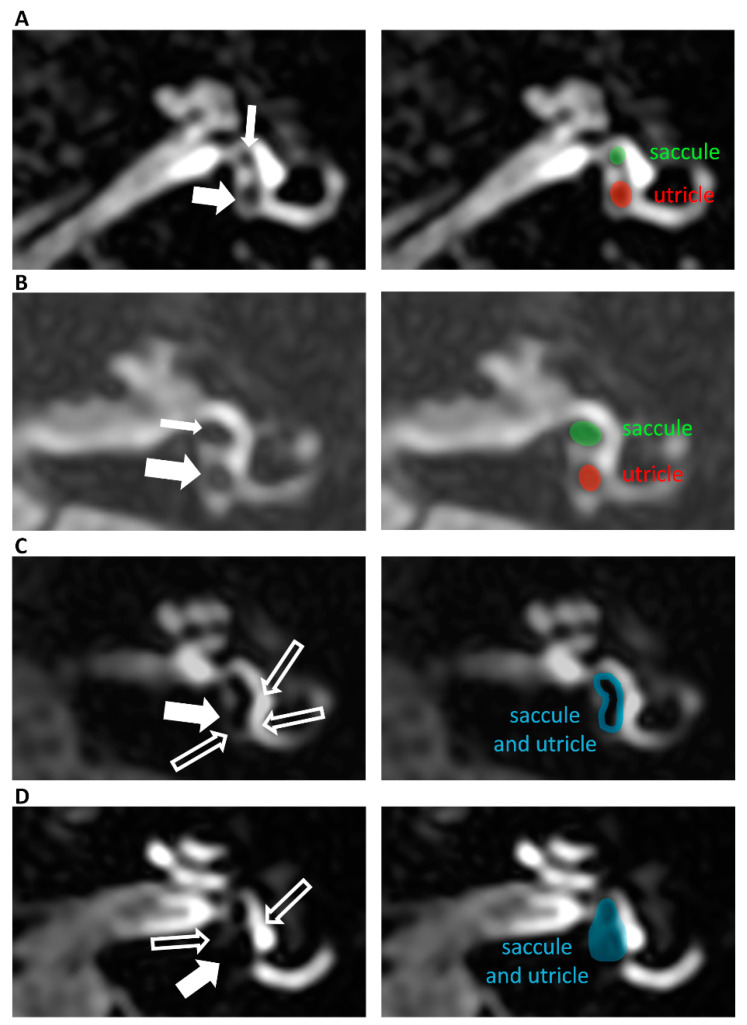
Delayed post-contrast 3D-FLAIR axial images of left ears below the mid-modiolar level (inferior part of the vestibule). The right side images on panel (**A**–**D**) show the same images as the left ones in respective panels but in the form of companion scans with some line art applied to define specific anatomical structures for easier identification of observed pathologies. Panel (**A**) presents a normal vestibule—grade 0 where non-enhancing the saccule (thin arrow) and utricle (thick arrow) are easily visible in the enhancing vestibule. The saccule is smaller than the utricle. Panel (**B**) presents extra low-grade, that is, grade 1 vestibular endolymphatic hydrops on Bearnaerts scale (but still grade 0 on Barath scale) where the saccule (thin arrow) and utricle (thick arrow) are well separated; however, the saccule is larger than the utricle, and the enhancing vestibule is seen around them. Panel (**C**) shows grade 2 vestibular endolymphatic hydrops on Bearnaerts scale (grade 1 on Barath scale) where the saccule and utricle are enlarged and confluent (thick arrow); however, thin enhancing boundaries of the vestibule are seen around them (long “empty” arrows). Panel (**D**) shows grade 3 vestibular endolymphatic hydrops on the Bearnaerts scale (grade 2 on the Barath scale), where significant enlargement of the saccule and utricle is so pronounced that the vestibule is almost totally obliterated (thin “empty” arrow). The saccule and utricle are enlarged and confluent (thick arrow); however, thin enhancing boundaries of the vestibule are seen around them (thin “empty” arrows).

*Vestibular endolymphatic hydrops on the Barath scale*, like cochlear endolymphatic hydrops, have been defined with a three-point scale (Figure 2). Grade 0—normal—means that the saccule and utricle are visible as nonenhanced structures within an enhanced vestibule. Grade 1 means some enlargement of the saccule and utricle, meaning that those structures are confluent and only the enhancing boundaries of the vestibule are still visible. Grade 2 indicates a significant enlargement of the saccule and utricle, so that the vestibule is obliterated.

In *vestibular endolymphatic hydrops in Bernaerts’ modification* of the Barath scale [8], a new grade, 1, was added, making the scale a four-point, from 0 to 3 (Figure 2). In the Bernaerts’ modification scale, grade 1 indicates small vestibular hydrops where the saccule is larger than the utricle but is still visually separated. Adding the new grade 1 means that grade 1 of the Barath scale becomes grade 2 and grade 2 becomes grade 3 in the modified Bernaerts scale.

**Figure 3:**
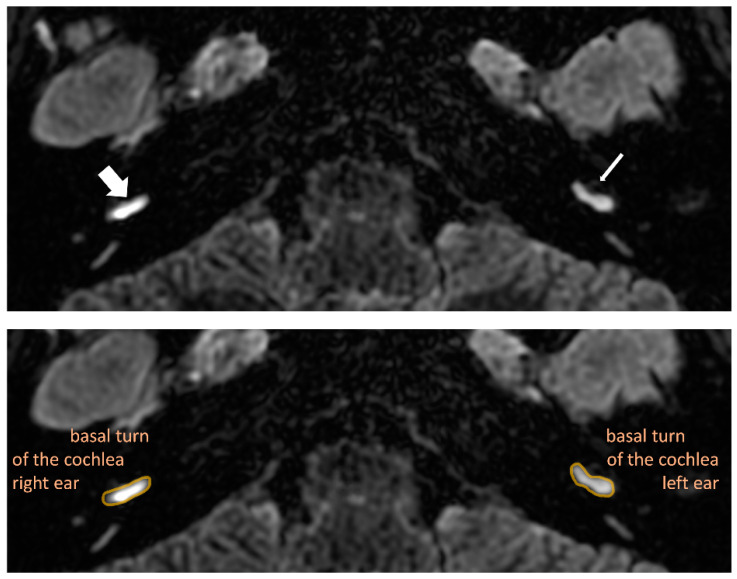
Delayed post-contrast 3D-FLAIR axial image of both ears at the level of the basal turn of the cochlea. In the top image, there is more robust enhancement of the basal cochlear turn on the affected right side (thick arrow), compared with normal perilymphatic enhancement on the left side (thin arrow). The bottom image is the same as the top one but in the form of a companion scan with some line art applied to define specific anatomical structures for easier identification of observed pathologies.

*Perilymphatic enhancement* describes the enhancement of the inner ear structures. In MD, it has been observed that perilymphatic enhancement is more robust on the affected side than on the healthy side [8,9,10] (Figure 3). The scale, in this case, consists of 2 stages, from 0 to 1, with 1 indicating a more robust enhancement, while 0 describes a regular enhancement.

Furthermore, the four features were analyzed in detail. The sensitivity and specificity of each feature to identify Ménière’s disease on MRI were calculated separately. Then the features mentioned above were combined to calculate the method’s overall sensitivity and specificity.

### 2.4. Statistical Analysis

Statistical analysis was carried out in STATISTICA software (StatSoft, Inc. 2017 analysis software system, version 13.3). The data were tested for normality, parametric and non-parametric criteria. Detailed statistical analysis was performed with the following tests: Friedman analysis of variance (ANOVA), Kendall’s concordance coefficient (Kendall’s W), and Wilcoxon signed rank. The level of statistical significance was set at *p* = 0.05.

## 3. Results

### 3.1. Results

#### 3.1.1. Patients’ Characteristics

The mean age of the patients at the time of MRI evaluation was 47.65 years old (range 20–84). There was a slight female predominance, with 64 females and 46 males.

Out of 110 patients, 72 were diagnosed with definite MD, 5 with probable MD, and 33 with other diseases and symptoms that do not fulfill the diagnostic criteria for MD. Of the patients with definite MD, 69 presented unilateral disease, and three bilateral (which equalled made 75 definite MD ears). Furthermore, among patients with unilateral definite MD, 15 had unspecified symptoms from the contralateral ear that do not fulfill diagnostic AAO–HNS criteria for MD. A total of 220 ears were evaluated in this study, of which 75 were definite MD ears, five probable MD ears, 67 ears with other Menieriform symptoms, and 73 were asymptomatic ears (Table 1).

#### 3.1.2. MRI Findings—Radiological Features Analysis

**Figure 4:**
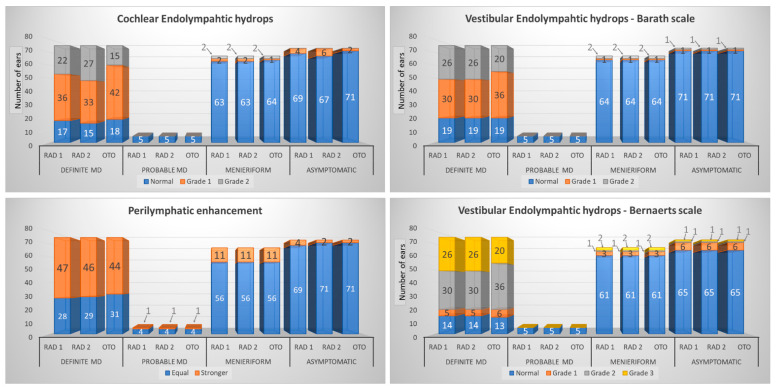
Stacked bar chart presenting differences of cochlear endolymphatic hydrops grading, vestibular endolymphatic hydrops grading on Barath scale, vestibular endolymphatic hydrops grading on Bernaerts modification, and perilymphatic enhancement assessment in each group of analyzed ears (definite Ménière’s, probable Ménière’s, ears with Menieriform symptoms, asymptomatic ears) performed by three observers (two radiologists and one otorhinolaryngologist in training; Rad1, Rad2, and Oto, respectively).

Cochlear endolymphatic hydrops. Within the group of definite MD ears, CoEH was present in 76–80% of ears, depending on the observer (Table 1). The sensitivity and specificity for CoEH diagnosis ranged between 0.76–0.8 and 0.92–0.97, respectively. In probable MD ears, CoEH was not observed. In patients with other Menieriform symptoms, CoEH was observed in 4.5–6%. CoEH was reported in 2.7–8.2% of asymptomatic ears, depending on the observer. The differences in CoEH grading are presented in Figure 4. The difference in observations was 5% between the two radiologists and 8% and 13% between radiologists (Rad1 and Rad2) and the otorhinolaryngologist, respectively. Multiple comparisons indicated significant differences between all three observers, confirmed by the low agreement value of Kendall’s W test (Table 2). Pairs of observers’ comparison showed significant differences, with a more considerable difference between the pairs of radiologists and otorhinolaryngologist. The main inconsistency was in the proper grading of CoEH, not in assessing the presence of any hydrops within the cochlea.

Vestibular endolymphatic hydrops on Barath scale. Using the qualitative Barath scale, VEH was observed in 75% of definite MD ears for all observers (Table 1). In asymptomatic ears, VEH was present in 2.7% of ears of patients with definite MD. The sensitivity for VEH was good at 0.75, and the specificity was very high at 0.97. In probable MD ears, VEH was not observed. In patients with other types of vertigo, EH was present in 4.5%. The differences in VEH grading are shown in Figure 4. The assessment of VEH on the Barath scale, comparing both radiologists, was precisely the same, and the only statistically significant differences were present between the pair of radiologists and the otorhinolaryngologist (Table 2). The main inconsistency was in the proper grading of VEH, not in assessing the presence of any hydrops within the vestibule.

Vestibular endolymphatic hydrops in Bernaerts’ modification of Barath scale. Adding one grade (extra-low VEH) to the Barath scale increased the frequency of VEH diagnosis in MD ears to 81.3–82.7% and in asymptomatic ears to 11% (Table 1). Consequently, it improved the sensitivity to 0.81–0.83 but slightly decreased the specificity to 0.89. In probable MD ears, VEH was not observed. In patients with other types of vertigo, VEH was present in 9% of ears. The differences in VEH grading are summarized in Figure 4 and Table 2.

Perilymphatic enhancement. An increased PE was observed in 58.7–62.7% of definite MD ears, 20% of probable MD ears, 16.4% of ears with other Menieriform symptoms, and 2.7–5.5% of asymptomatic ears (Table 1). The differences in perilymphatic enhancement assessment are shown in Figure 4. The ANOVA Friedman test showed no significant differences for this parameter between the assessment of all three observers and between the pairs of observers (Table 2).

The above-mentioned combined features to identify Ménière’s disease on MRI (endolymphatic hydrops (both cochlear and vestibular) and increased perilymphatic enhancement) gave a sensitivity ranging between 0.84 and 0.87 and specificity of 0.82–0.88.

## 4. Discussion

In the last few years many MRI studies have been performed on MD but, still, there is a lack of a gold standard for MRI protocols and assessment methods. Different protocols of examinations (both methods of contrast administration and sequences employed on MRI), various scales, and biomarkers are used in radiological diagnosis. Some researchers have used intratympanic [4,11,12,13,14,15,16,17], others intravenous administration of gadolinium-based contrast-agent [6,8,18,19,20,21]. Yamazaki et al. [22] compared two methods of contrast injection and suggested that the intratympanic contrast administration provides better perilymphatic enhancement than intravenous and probably should be used in unilateral MD. However, the intratympanic method is invasive, allows for unilateral examination only, is off-label GBCA use, and requires 24-h waiting time [18,23], so the intravenous method is widely used as more feasible and comfortable for the patient. Furthermore, this method of contrast administration allows the assessment of EH and blood–perilymph barrier permeability.

The two principal sequences applied to EH imaging are 3D-FLAIR and 3D-REAL IR, compared visually with the heavily T2 cisternography sequence for anatomical reference. Furthermore, Naganawa et al. developed a series of subtraction sequences such as HYDROPS [23], HYDROPS2 [24], and Hydrops-Mi2 [25], but those techniques are still used as clinical research methods and are not commonly available. Moreover, there are two main EH grading methods, and each evaluates cochlear and vestibular EH separately. Some researchers have used the semiquantitative scale proposed by Nakashima [5], while others have used the qualitative scale described by Barath et al. [6]. In addition, the latter has been recently modified by adding a grade to the VEH evaluation [8]. Each of the factors mentioned above can affect image quality and final assessment.

Furthermore, in some studies, the diagnosis of EH was made by one author [26], or if more observers were involved, the diagnosis of EH was made by consensus [4,23,27,28] or there is a lack of information about the observers’ agreement [29,30,31,32]. Consequently, the reported frequency of EH in MD and asymptomatic ears varies in the literature, and debate still exists if the EH, especially CoEH, is a valuable biomarker of MD [7].

Our study aimed to evaluate if the discrepancy in the prevalence of EH in MD might result from the radiological criteria used. We wanted to investigate if the criteria, such as assessment of the presence of EH in the Barath scale and Bernaerts’ modification of this scale and increased perilymphatic enhancement, are repeatable and easy to evaluate. For this purpose, the assessment of MRI examinations of all the ears in our group was made by two experienced radiologists and one otorhinolaryngologist in training.

Our study showed that the frequency of cochlear endolymphatic hydrops in definite MD ears was 76–80%. This type of endolymphatic hydrops was more often detected by experienced observers (Rad 1 and 2) than by the observer in training (Oto). Our results can be compared with other studies based on the same MRI sequence—delayed post-contrast 3D-FLAIR. In Barath et al.’s [6] study, this type of hydrops was described in 86.9% of MD ears, van Steekelenburg et al. [21] reported the presence of CoEH in 85% of definite MD ears, and Pai et al. [30] recognized CoEH in 100% of MD ears. In authors that used other MRI sequences (most often three dimension inversion recovery sequence—3D-IR), the frequency of CoEH in MD patients was 90% in Shi et al. [33], while Ito et al. [28] reported this biomarker in 62% of MD ears, and Yoshida et al. [34] observed CoEH in 87% of MD ears. Suárez Vega et al. [27] compared 3D-FLAIR and 3D-IR and found CoEH in 75% of definite MD ears. In our study, in a group of ears without a diagnosis of MD, CoEH was rare, ranging from 4.5 to 6% for ears with Menieriform symptoms and 2.7–8.2% for asymptomatic contralateral ears in MD patients. Similar results were described by van Steekelenburg et al. [21]. They observed CoEH in 3.1% of ears with Menieriform symptoms and 2% of asymptomatic ears MD patients. Ito et al. [28] observed this in 6.3% of asymptomatic ears.

In our study, all the observers detected vestibular endolymphatic hydrops using the Barath scale in 74.7% of definite MD ears. Referring to other studies that used the 3D-FLAIR sequence, our results are similar to those reported by Paskoniene et al. [29], who revealed VEH in 76.4% of MD ears, and lower than results obtained in studies by Barath et al. [6] (92%), van Steekelenburg [21] (89%) and by Pai et al. [30] (86%). Similar results were obtained with other MRI sequences, as follows: Shi et al. [33] described VEH in 88% of MD ears, Ito et al. [28] in 66%, and Yoshida et al. [34] in 94%. Suárez Vega et al. [27] described VEH in 92% of MD ears. In our study, for ears with other symptoms that do not fulfil the criteria for MD but presented some symptoms, VEH was assessed only in 4.5%, and similarly in asymptomatic contralateral ears in 2.7%.

In our study, adding one grade to Barath’s classification of vestibular endolymphatic hydrops, as was described by Bernaerts, changed the frequency of VEH identification in definite MD ears to 81–83%, depending on the observer. However, this criterion changed the frequency of recognizing VEH in ears with Menieriform symptoms to 7.5% and 11% in asymptomatic ears. Consequently, the sensitivity increased, but the specificity decreased. Similarly, in Bernaerts et al.’s study [8], this feature increased the sensitivity of VEH from 79.5% to 85%, and in the study of Jasinska et al. [10], from 82% to 92%. It is worth emphasizing the extra low VEH in asymptomatic ears of six patients diagnosed with unilateral definite MD and two with sudden deafness. This finding might be explained by the fact that MD is often a bilateral disease with different onset in each ear, and dilation of the saccule might be an early sign of MD before symptoms appear [10,35,36].

In the literature, increased perilymphatic enhancement of the inner ear structures as a sign of blood–perilymph barrier impairment was described as the next probable biomarker of MD [6,8,21,22,37,38,39]. Our study observed it in 59–63% of definite MD patients, in the group of ears with other symptoms in 13%, and only 2.7% in asymptomatic ears. In Bernaerts et al.’s [8] study, 67.9% of MD patients had increased PE on the affected side. Van Steekelenburg et al. [21] observed increased PE in 82.6% of MD, 9.4% of Menieriform symptoms, and 3.4% of asymptomatic ears. However, this parameter should probably be combined with the EH because it may also be present in other ear diseases, such as sudden sensorineural hearing loss or vestibular neuritis [21,40].

Ménière’s disease is a complex disease with heterogeneous symptoms. Moreover, it is chronic and gradually progresses from monosymptomatic to fully symptomatic. The actual diagnostic criteria (AAO–HNS) for diagnosing MD include patients with advanced-stage disease. Therefore, the diagnosis of this disease is often difficult. According to the literature, in 20% of patients, it takes more than five years to diagnose MD [41]. Additionally, it was discovered that the presence of EH may precede symptoms in MD patients [8,41], can progress with the disease duration, and is correlated with clinical symptoms [10,42,43,44,45,46,47,48]. Therefore, it can serve as a method of early detection and support the diagnosis in clinically atypical cases, help to choose proper treatment, and potentially monitor therapeutic effects [39,47,49,50].

In our study, when assessing the radiological features, the highest differences between the observers occurred for the evaluation of cochlear endolymphatic hydrops. A significant difference was found between all pairs of observers, with a more considerable difference between the pairs of radiologists and otorhinolaryngologist. Earlier studies suggest that the assessment of CoEH on delayed post-contrast 3D-FLAIR sequence might be interpreted variably [27,51,52]. Our study confirmed this finding; however, it also showed 96% interobserver agreement for the differentiation of hydropic and normal ears. Consequently, it showed that it is much easier to classify ears as normal or hydropic than to choose the proper grade of endolymphatic hydrops.

The assessment of vestibular endolymphatic hydrops in both Barath and Bernaerts scales, comparing both radiologists, was precisely the same. The only differences were present between radiologists and otorhinolaryngologist when assessing the VEH on the Barath scale, not on the Bernaerts scale. However, like with CoEH, the main non-accordance was in the proper grading of hydropic ears, not in assessing the presence of any hydrops. All observers differentiated normal and hydropic ears almost the same way. When comparing interobserver agreement, our results slightly differ from the literature. Most researchers showed a higher concordance coefficient for CoEH than VEH recognition, as follows: Barath et al. [6], 0.97 for CoEH and 0.94 for VEH grading, van Steekelenburg et al. [21], 0.93 for CoEH and 0.92 for VEH, Bernaerts et al. [8], 0.83 for CoEH and 0.81 for VEH. However, Suárez Vega et al. [27] reported that degree of concordance was higher for VEH (0.66) than for CoEH (0.39) using the 3D-FLAIR sequence. Moreover, they also found that the concordance coefficient was higher (0.82) when diagnosing any EH than scoring it. The latter is similar to our findings.

As for the perilymphatic enhancement of the inner ear structures, there were no significant differences between the three observers and the pairs of observers for this parameter, which is in line with the literature [8,21].

In our study, three observers evaluated MRI scans independently to evaluate if the assessment method was repeatable. By engaging a less experienced MRI observer (otorhinolaryngologist) who was trained with a few cases of MRI scans of inner ears, the study aimed to evaluate if the method was easy to learn. It showed that the assessment of vestibular endolymphatic hydrops is easy to learn and repeatable. For experienced radiologists, the readings for 220 ears were the same, whereas, for the otolaryngologist, the number of different observations was very low. Typically, the saccule and utricle are easily identified in the vestibule’s inferior part as two “black dots” surrounded by a bright (enhancing) rim of perilymph. Furthermore, the saccule is smaller than the utricle. Even for an untrained observer, it is not difficult to find that this configuration is changed. That is probably the reason for such high agreement in assessing this parameter.

Similarly, with the perilymphatic enhancement qualitative assessment, it is not difficult to visually compare two sides and find an asymmetry.

When it comes to cochlear endolymphatic hydrops assessment using a 3D-FLAIR MRI sequence, this is more complicated. In this sequence, differentiating endolymphatic structures from surrounding bone is impossible because both have low signals [27]. First, changes in the contours of the cochlea should be found and compared with the FIESTA sequence as an anatomic reference. Second, how much outlines are changed should be evaluated, and then CoEH should be graded. Evaluation of this parameter is more complicated, which is probably why it is so difficult to properly stage EH, even for experienced users. According to existing literature, the solution to this problem might be using a post-contrast 3D-IR inversion recovery sequence [23,24,27,53]; however, this sequence is not widely available, takes longer, and is more prone to motion artifacts. The repeatability of CoEH assessment using different sequences needs to be confirmed in further studies.

## 5. Conclusions

Our study confirmed that endolymphatic hydrops and robust perilymphatic enhancement are much more often present in MD ears than in other inner ear diseases. Both cochlear and vestibular endolymphatic hydrops parameters may serve as a method of differentiation of MD from normal ears. It showed that evaluation of vestibular endolymphatic hydrops is repeatable between observers and easy to learn. Furthermore, it proved that Bernaerts’ modification increased the sensitivity of endolymphatic hydrops diagnosis. It also confirmed the recent opinion that it is more challenging to assess cochlear than vestibular endolymphatic hydrops using an MRI 3D-FLAIR sequence. The interpretation of MRI is more consistent when evaluated by radiologists in the case of cochlear hydrops. In addition, when assessing cochlear hydrops, distinguishing between normal and hydropic ears is much easier to perform than EH grading. Therefore, it may be used to diagnose MD rather than EH staging. These observations could shed new light on the usefulness of MRI 3D-FLAIR in diagnosing cochlear endolymphatic hydrops.

The method of EH diagnosis using MRI scans described above is not difficult to perform, and it may be included in the diagnostic protocol of MD to support the diagnosis of MD in clinical settings.

In conclusion, the most important findings of this study for otolaryngologists are as follows:

1. Visualization of endolymphatic structures is possible.
2. Endolymphatic hydrops is present more often in Ménière’s disease ears than in other inner diseases and much more frequent than in asymptomatic ears, so it might be used as a diagnostic criterion to support the diagnosis of Ménière’s disease for clinically unclear cases.
3. Increased perilymphatic enhancement is not difficult to assess and is present more often in Ménière’s disease than in other inner ear pathologies.
4. Quick diagnosis of endolymphatic hydrops on MRI is not difficult, even for quickly trained observers, that is, otorhinolaryngologists after a short period of training.

In conclusion, the most important findings of this study for radiologists are as follows:

1. The assessment of vestibular hydrops on the two scales mentioned above is easy to learn. The modified scale increases the sensitivity and specificity of the method to diagnose Ménière’s disease ears, so it can be used for diagnosing and staging vestibular endolymphatic hydrops.
2. Evaluation of cochlear hydrops is more complicated than vestibular. However, the main concern is not to diagnose pathology but to grade it properly (which means to answer the question “is it 1st or 2nd grade endolymphatic hydrops?”) rather than to diagnose it (“is it normal = 0 or abnormal = 1st or 2nd grade?”). Therefore, it should be used for the diagnosis of endolymphatic hydrops, not for grading it.

## Figures and Tables

**Table 1:** The frequency of endolymphatic hydrops and increased perilymphatic enhancement of the inner ear structures in analyzed ears evaluated by three observers (two radiologists and an otorhinolaryngologist in training).

		CoEH Number of Ears (Percentage)			VEH Barath Number of Ears (Percentage)			VEH Bernaerts Number of Ears (Percentage)			PE Number of Ears (Percentage)		
**Clinical diagnosis**	**Number of ears** (total 220)	Rad1	Rad2	Oto	Rad1	Rad2	Oto	Rad1	Rad2	Oto	Rad1	Rad2	Oto
**Definite MD**	75	58 (77%)	60 (80%)	57 (76%)	56 (74.7%)			61 (81.3%)		62 (82.7%)	47 (62.7%)	46 (61.3%)	44 (58.7%)
**Probable MD**	5	0 (0%)									1 (20%)		
**Other diseases**	67	4 (6%)	4 (6%)	3 (4.5%)	3 (4.5%)			5 (7.5%)			9 (13.4%)		
**Asymptomatic**	73	4 (5.5%)	6 (8.2%)	2 (2.7%)	2 (2.7%)			8 (11%)			4 (5.5%)	2 (2.7%)	

CoEH—cochlear endolymphatic hydrops (grades 1, 2 together); VEH Barath—vestibular endolymphatic hydrops in scale proposed by Barath; VEH Bernaerts—vestibular endolymphatic hydrops in Bernaerts’ modification of the Barath scale; PE—increased perilymphatic enhancement; MD—Ménière’s disease; Rad1—radiologist #1; Rad2—radiologist #2; Oto—otorhinolaryngologist in training.

**Table 2:** Statistical dependencies (*p*-values) of ANOVA Friedman test, Kendall’s concordance coefficient, and Wilcoxon signed-rank test—evaluation of each analyzed MRI hydrops feature differences across three observers (two experienced radiologists and otorhinolaryngologist in training) to assess inter-judge reliability.

Radiological Feature	Friedman ANOVA *p*-Value	Kendall’s Concordance Coefficient	Pairs of Observers Compared	Number of Different Observations	Wilcoxon Test Results *p*-Value	ANOVA Test Results *p*-Value
**CoEH**	0.0000 *	0.0456	Rad1 vs. Rad2	11	0.0164	0.0067 *
			Rad1 vs. Oto	19	0.0269	0.0116 *
			Rad2 vs. Oto	30	0.0014	0.0003 *
**VEH Barath**	0.0111 *	0.0205	Rad1 vs. Rad2	0	--	--
			Rad1 vs. Oto	8	0.0587	0.0339 *
			Rad2 vs. Oto	8	0.0587	0.0339 *
**VEH Bernaerts**	0.1030	0.0103	Rad1 vs. Rad2	0	--	
			Rad1 vs. Oto	11	0.1823	0.1317
			Rad2 vs. Oto	11	0.1823	0.1317
**PE**	0.2053	0.0072	Rad1 vs. Rad2	7	0.3105	0.2568
			Rad1 vs. Oto	11	0.1823	0.1317
			Rad2 vs. Oto	6	0.4631	0.4142

The asterisk (*) and bold font were used to mark statistically significant differences (*p* < 0.05). CoEH—cochlear endolymphatic hydrops (grades 1, 2 together); VEH Barath—vestibular endolymphatic hydrops in scale proposed by Barath; VEH Bernaerts—vestibular endolymphatic hydrops in Bernaerts’ modification of the Barath scale; PE—increased perilymphatic enhancement; Rad1—radiologist #1; Rad2—radiologist #2; Oto—otorhinolaryngologist in training.

## Data Availability

Not applicable.
